# Effects of Different Nutritional Conditions on the Growth and Reproduction of *Nilaparvata lugens* (Stål)

**DOI:** 10.3389/fphys.2021.794721

**Published:** 2022-01-04

**Authors:** Kui Kang, Youjun Cai, Lei Yue, Wenqing Zhang

**Affiliations:** ^1^Key Laboratory of Regional Characteristic for Conservation and Utilization of Zoology Resource in Chishui River Basin, College of Biology and Agriculture, Zunyi Normal University, Zunyi, China; ^2^State Key Laboratory of Biocontrol and School of Life Sciences, Sun Yat-sen University, Guangzhou, China

**Keywords:** Delphacidae, nutritional deficiency, energy distribution, developmental period, fecundity

## Abstract

Growth and reproduction are the two most basic life processes of organisms and the distribution of energy in these processes is a core issue of the life history of organisms. *Nilaparvata lugens* (Stål), the brown planthopper (BPH), is a single-feeding rice pest. In the present study, this species was used as a model for testing the effects of nutritional conditions on various growth and reproduction indicators. First, the third-instar nymphs were fed with three different concentrations (100, 50, and 25%) of artificial diet until the second day of adulthood. The results showed that as the nutrient concentration decreased, the body development and oviposition of BPH were hindered. The total lipid content in the fat bodies was also significantly reduced. RT-PCR analysis showed compared to the 100% concentration group, the expression levels of *vitellogenin* (*Vg*) genes in the fifth-instar nymphs, adults, and in different tissues (ovary, fat body, and other tissues) were significantly decreased in the 50 and 25% treatment groups. Western blot analysis showed that Vg protein expression was highest in the 100% group, followed by the 50% group, with no expression in the 25% group. These results indicate that growth and reproduction in the BPH are regulated by, or correlated with, nutrient concentration. This study is of great significance as it reveals the adaptive strategies of the BPH to nutritional deficiencies and it also provides valuable information for the comprehensive control of this pest.

## Introduction

Diseases and pests affecting the yield of rice in China hinder the country’s long history of rice cultivation. *Nilaparvata lugens* (Stål), the brown planthopper (BPH), is a major migratory rice pest that had outbreaks in China for three consecutive years, from 2005 to 2007, with an annual damage area of 26.67 million hectares and a direct economic loss of 15 billion yuan (Cheng et al., 2013). BPH eggs are banana-shaped and are generally found on the rice leaf’s sheath. The species’ nymphs are oval and present five instars. Adults have lustrous dark or light brown dorsal parts and long or short wings (Fowler et al., 1991). Nymphs and adults typically cluster at the lower part of the rice stem, where they grow and develop by sucking rice stem juice. Moreover, their feeding and oviposition behaviors can damage stem and leaf tissues, accelerate withering or lodging, and induce the appearance of diseases, such as the rice yellow dwarf and rice sheath blight diseases (Fowler et al., 1991; Li et al., 2017). Therefore, an effective control of BPH improves the yield and quality of rice.

Insects have high nutritional and energy requirements to maintain their growth, development, and reproduction, so the primary factor of insect population outbreaks is to provide adequate food (Behmer and Joern, 2012). The free amino acid content of rice plants plays an important role in the development of BPH, which they directly obtain from rice juice (Yue et al., 2018). The demands for proteins and carbohydrates change throughout the development cycle of insects (Liu et al., 2017). For example, female insects have been shown to mainly require proteins during the oviposition period and carbohydrates after the oviposition period (Simmonds et al., 1992; Ma et al., 2020). Furthermore, compared to the long-flying locust, the oriental migratory locust is more inclined to carbohydrate uptake (Chambers et al., 1995). However, often food shortages and population booms result in nutritional deficiencies. Nevertheless, food shortages do not necessarily result in death as insects normally adopt different strategies to resist nutrient deficiency, depending on the insect group (McCue, 2010). The main strategies include as: (1) storing more energy, (2) reducing energy consumption, (3) moving to better habitats, and (4) altering energy distribution in the body (Kooijman, 2009; Rion and Kawecki, 2010). Previous studies on insect longevity and reproduction have pointed out that the balance of protein and non-protein energy intake is a key factor (Simpson and Raubenheimer, 2007; Solon-Biet et al., 2014). It has also been revealed that nutritional balance significantly affects the fecundity of adult insects (Warbrick-Smith et al., 2006; Simpson and Raubenheimer, 2009). The reduction of diets can often prolong an insects’ life span, but simultaneously reduce vitality and health (Dobson et al., 2018).

The lack of nutrition at the nymph stage is assumed to affect the physiological balance between the growth and reproduction of female *N. lugens*. Therefore, in the present study, the effect of different feed concentrations on the growth and reproduction of BPH nymphs was tested. This study will lay a foundation for further studies on the molecular mechanisms of the effects of nutrition on the growth and reproduction of BPH.

## Materials and Methods

### Experimental Materials

The non-resistant rice variety, Huanghuazhan, collected from Shaoguan, Zhaoqing, Huizhou, and Qingyuan, and maintained in a mixed culture in the laboratory for nearly four years was used for breeding the BPH. Feeding conditions were as follows: a temperature of approximately 28°C, a humidity range of 80–90%, and a light cycle of 14:10 h (light:dark). Newly hatched third-instar nymph was used in this experiment.

### Preparation and Feeding With Artificial Diet

Full chemical artificial diet D-97 was prepared following the method of Fu et al. (2001). Sterile water was used to add to a volume of 100 ml at a pH of 6.8. Total pure D-97 artificial diet was used as the basic 100% concentration diet, the 50, and 25% concentration diet were prepared by diluting the basic 100% concentration diet to keep the proportion and physical and chemical properties of other components unchanged. The feeding method used as previous (Qiu et al., 2016). Twenty third-instar nymphs were placed in each chamber as replicates, and we performed the experiments in triplicate for each concentration diet.

### Determination of the Growth State

Twenty 3rd-instar nymphs were placed in separate tubes according to the treatment. Daily survival rate, body weight, and body length were recorded on the second day after eclosion as well as the survival rate and development period. Ovaries were dissected and evaluated for ovarian development stage (Lu et al., 2011).

### Quantitative Real-Time PCR Analysis

Total RNA was extracted from different age groups (2-day-old fourth and fifth-instars nymphs, adult) and tissues (ovary, fat body, and among others). RNA was prepared from each tissue or the whole body using the TRIzol method (Invitrogen) following the manufacturer’s protocol, and treated with DNase I (Takara Bio). RNA quantity was evaluated using a microvolume spectrophotometer (NanoDrop 2000, Thermo). For this assay, 1 μg of RNA was used for first-strand cDNA synthesis with the PrimeScript^™^ 1st Strand cDNA Synthesis Kit (Takara Bio, Japan). The primers used for real-time PCR are listed in Supplementary Table S1. The cDNA was amplified by PCR in 10 μl reaction mixtures using the Light Cycler 480 system (Roche Diagnostics, Indianapolis, IN) and SYBR Premix Ex Taq (Roche) using the following procedure: 94°C for 5 min, followed by 45 cycles of 94°C for 15 s, 60°C for 20 s, and 72°C for 15 s. The β-actin gene was used as an internal standard (Kang et al., 2018). After the amplification protocol, a melting curve analysis was performed in triplicate, and the results were averaged. The quantitative variation for each gene was calculated using a relative quantitative method (2^−ΔΔCT^) for three independent biological samples (Livak and Schmittgen, 2001).

### Western Blot

Fifteen specimens of each group of nymphs (2-day-old fourth and fifth instars nymphs and adult, respectively, and thirty 6-day-old adults) were randomly selected and divided into three groups based on the experimental tissues. The sample was lysed in 1 × Passive Lysis Buffer. The homogenate was centrifuged at 12,000 × *g* at 4°C for 15 min, and protein contents in the supernatant were measured by the Bradford method. The Western blot technique was modified according to previously described methods (Mitsumasu et al., 2008). In this study, 30 μg of total protein was separated on a 10% SDS–PAGE and transferred to poly (vinylidene difluoride) membranes (0.45 μm, Milli-pore), and the membranes were immunoblotted with anti-Vg (vitellogenin, 1:500, Abmart) and anti-β-actin (1:4,000, Abcam). Secondary antibody was immunoglobulin G (IgG) goat anti-rabbit antibody conjugated to horseradish peroxidase (HRP; 1,20,000, Trans; Chen et al., 2019). The membranes were visualized using electrochemiluminescence (Milli-pore) and Image Lab (Bio-Rad). The protein bands were quantified by importing the images into Image J analysis software (Wayne Rasband).

### Determination of Total Lipid Content in Fat Bodies

Fat bodies were dissected from 30 two-day-old BPH adults treated with 200 μl grinding buffer (100 mm KH_2_PO_4_, 1 mm DTT, and 1 mm EDTA), followed by centrifugation at 14,000 rpm for 10 min. The supernatant was transferred to a new centrifuge tube, and chloroform methanol solution was added for extraction (chloroform:methanol = 1:2). After a second centrifugation at 14,000 rpm for 10 min, the supernatant was used as the test solution. Thereafter, 200 μl of the solution was added to 50 μl of concentrated sulfuric acid; this was maintained in a water bath at 90°C for 2 min. Subsequently, 950 μl of vanillin phosphoric acid solution was added (to a final concentration of 1.2 g/l) and kept at room temperature for 20 min. Finally, the OD value was detected at the wavelength of 525 nm and the lipid content was calculated according to the standard curve of Triolein.

### Statistical Analysis

Protein expression levels were analyzed using the ImageJ software (Wayne Rasband, National Institutes of Health, United States). All the results were based on the mean values. The Duncan method was used to analyze the relative quantitative data of genes. Different letters above the same index data indicate that the difference is significant (*p* < 0.05).

## Results

### Effects of Nutrition on the Body Size and Body Weight of the BPH

**Figure 1:**
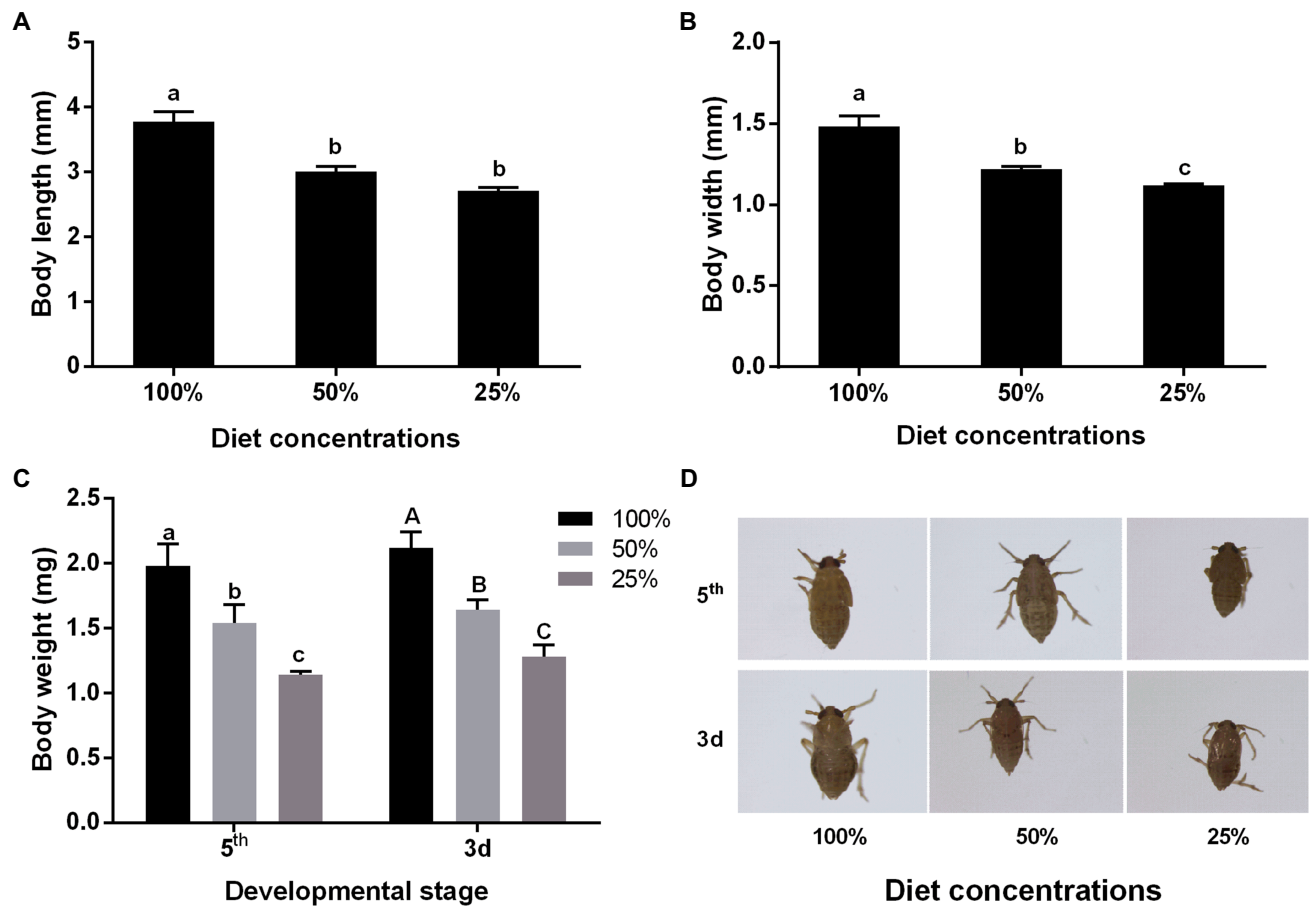
Effect of different nutrient concentrations on the body shape of the brown planthopper (BPH). **(A)** Body length; **(B)** Body width; **(C)** Weight (fifth: fifth-instar nymph; 3d: Adult of 3 day old); and **(D)** Phenotype (fifth: fifth-instar nymph; 3d: Adult of 3 day old). Data are presented as mean ± SE (*n* = 3); different letters above the same index data indicate significant differences (*p* < 0.05, Duncan’s multiple range test).

By measuring the body size and weight of insects, the results showed that low diet concentration could influence body speech. At 25% diet concentration, both the body length and width decreased by about 30% compared with 100% diet concentration (Figures 1A,B). The results of monitoring the body weight of insects showed that there was a significant decrease of about 40% in the low diet concentration, whether in nymph or adult stage (Figure 1C). And we also could see the difference in the photos (Figure 1D).

### Effects of Nutrition on the Survival and Development of the BPH

**Figure 2:**
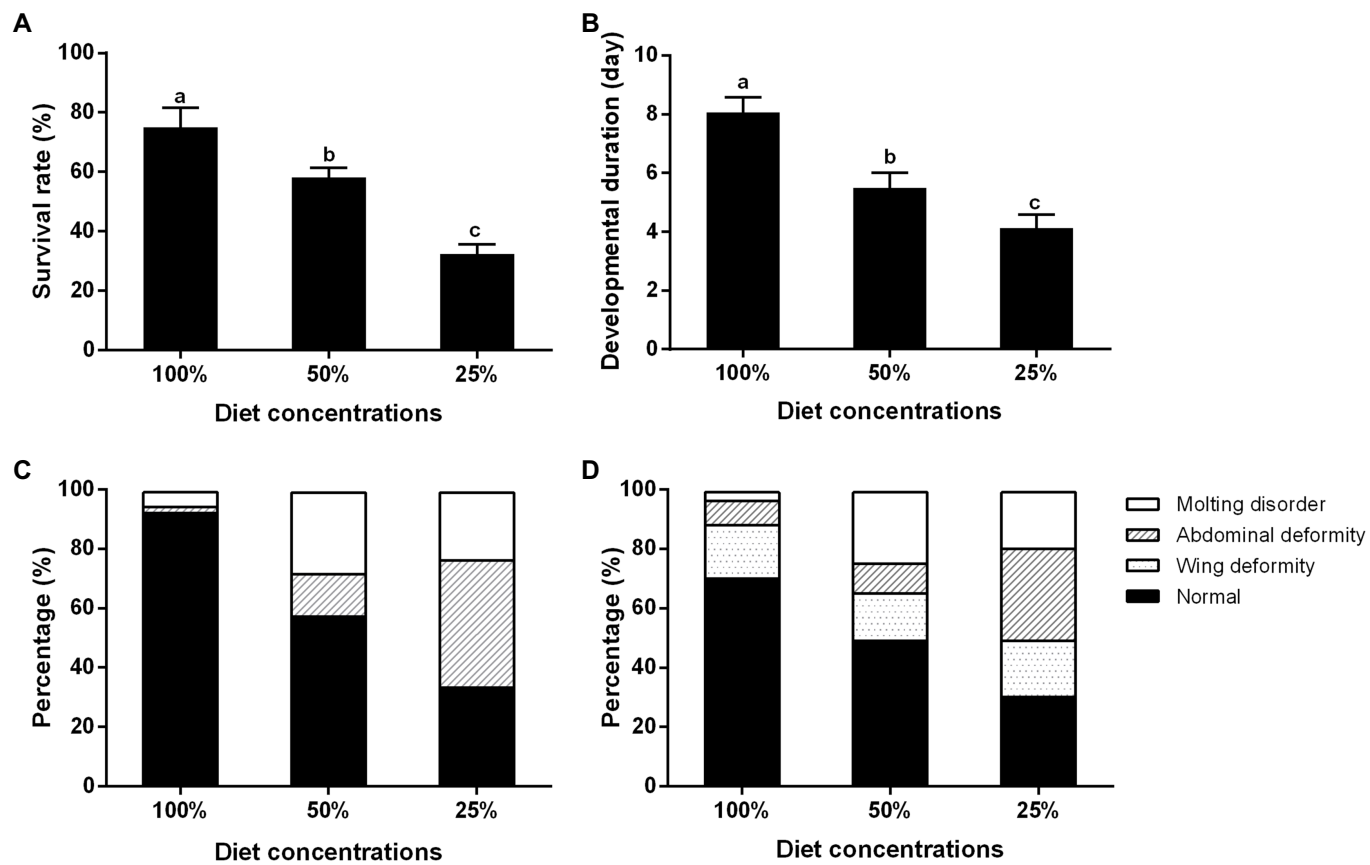
Effects of nutrition on the survival and development of the BPH. **(A)** Survival rate; **(B)** Developmental period; **(C)** Deformity ratio of the BPHs of fifth-instar nymph; and **(D)** Deformity ratio of the BPHs of adult of 2 day. Data are presented as mean ± SE (*n* = 3); different letters above the same index data indicate significant differences (*p* < 0.05, Duncan’s multiple range test).

With the decrease in diet concentration, the survival rate of the BPH significantly decreased from the two-day-old third-instar nymphs to the two-day-old adults (Figure 2A), and the developmental period of adults was significantly shortened (Figure 2B). Otherwise with the decrease in diet concentration, a variety of abnormal phenotypes appeared, such as variations in ecdysis, curled wings, and short or shriveled abdomens. At the 100, 50, and 25% diet concentrations, deformity accounted for about 10, 40, and 70% of the individuals, respectively. With the decrease in feed concentration, the proportion of abdominal deformities increased (Figures 2C,D).

### Effects of Nutrition on the Fecundity of BPH

**Figure 3:**
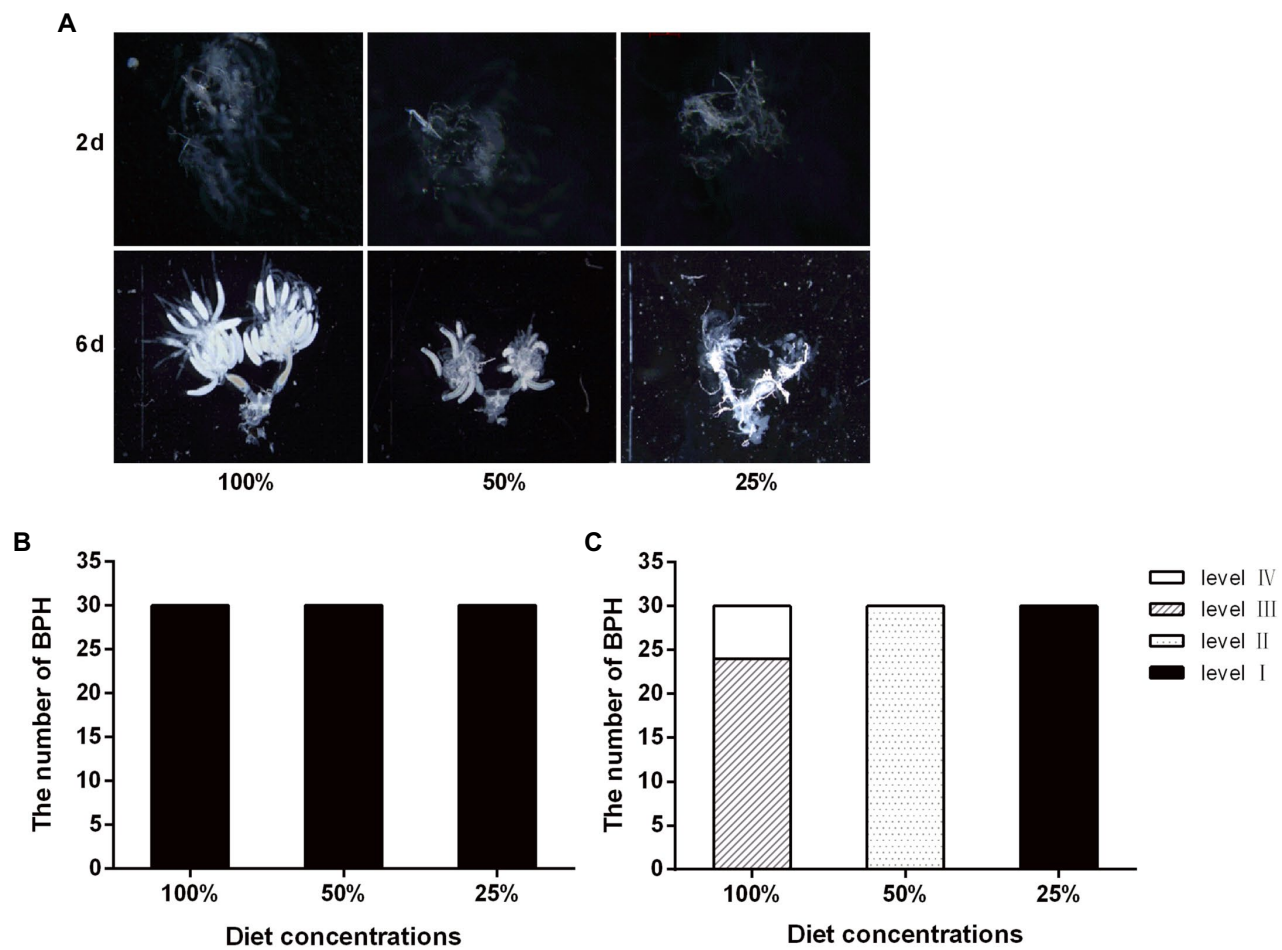
Effect of nutrient concentration on the ovary development of the BPH. **(A)** Ovaries of adults fed with different concentration diet for 2 day of adult (2d) and 6 day of adult (6d); **(B)** Ovarian grading of BPH on 2 day of adult; and **(C)** Ovarian grading of BPH on 6 day of adult.

According to the ovarian grading characteristics of BPH (Lu et al., 2011), the observation showed that the entire ovarian tissue was scattered, no mature eggs were formed, and no content precipitation was observed on the second day of feeding. On the sixth day of adult growth, the change in nutrient concentration affected ovary development. Under the 100% feed concentration treatment, most of the ovaries developed up to a third-level state, while the remaining ovaries developed up to a fourth-level state. Under the 50% feed concentration treatment, the ovarian development reached the secondary state. No significant difference in ovarian development was observed with the decrease in nutrition concentration. After 6 days of feeding, the entire ovarian tissue was completely developed, the ovarian tubule contents were clearly precipitated, and the number of mature eggs was higher than another two lower diet concentration. When fed with the 50% feed concentration treatment, the entire ovarian tissue was translucent and a small number of oocytes was formed at the inferior portion of the ovarian tubules. When fed with the 25% feed concentration treatment, the fallopian tube was translucent and no content precipitation was observed in the ovarian tubules; moreover, few eggs were formed, but no mature ones (Figure 3).

**Figure 4:**
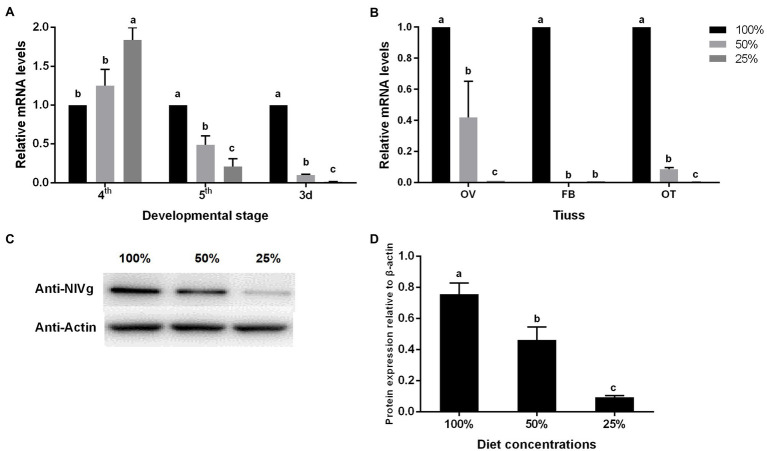
Effect of nutrient concentration on Vg expression of the BPH of **(A)** different ages, **(B)** different tissues, and **(C,D)** Vg protein accumulation of the BPH in adults on day 6. Data are presented as mean ± standard error (*n* = 3); different letters represent significant differences (*p* < 0.05, Duncan’s multiple range test).

The RT-PCR analysis was used for detecting the expression level of the *vitellogenesis* (*Vg*) gene, a molecular index of BPH fecundity. The results showed the expression level of *Vg* in the fifth-instar nymph, and on the second day of adulthood, significantly decreased under the low nutrient concentration treatment (Figure 4A). This also occurred in the *Vg* expression in the ovaries, fat bodies, and other tissues (Figure 4B). Vg protein accumulation in the ovaries of adults was detected continuously for 6 days, which is consistent with the morphological observations, and showed a positive correlation with nutrient concentration (Figures 4C,D).

### Effects of Nutrition on the Total Lipid Content of Liposomes in the BPH

**Figure 5:**
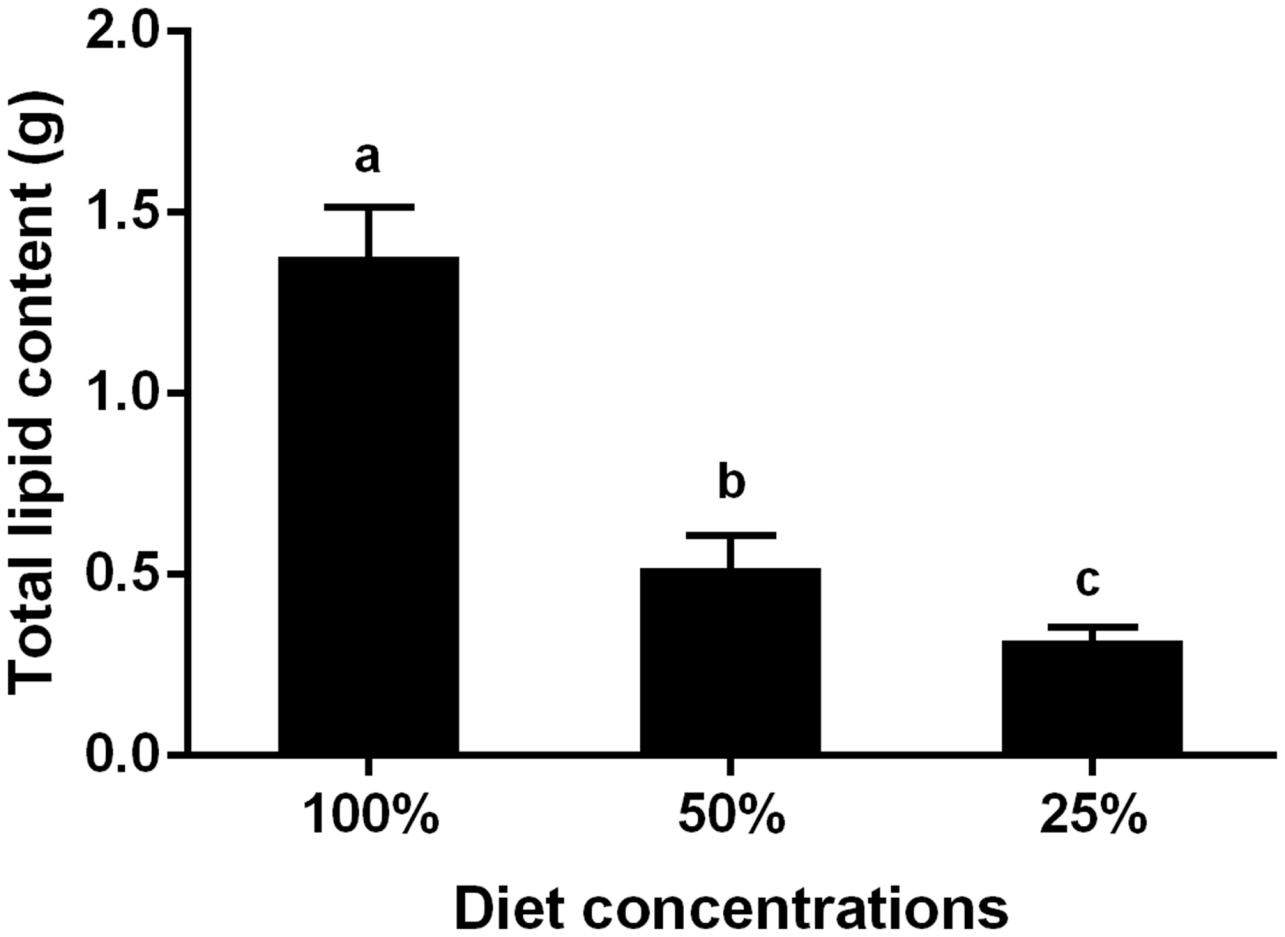
Effects of different nutrient concentrations on the total lipid content in six-day-old BPH adult. Data are presented as mean ± SE (*n* = 3); different letters represent significant differences (*p* < 0.05, Duncan’s multiple range test).

By detecting the lipid content in the fat bodies of BPH adults fed to day 6 under three conditions, we found fed by the 100% diet concentration was about 1.4 g, and there was 0.4 g that fed in 25% diet concentration conditions. The results showed that the total lipid content decreased significantly as well as the decrease of diet concentration (Figure 5).

## Discussion

Food is the main source of nutrients for insects. Long food shortages affect insect growth, development, reproduction, and may even cause death (Kang et al., 2011). Body size, weight, survival rate, and deformity rate are often used as indicators of growth status under nutritional deficiency (Gergs et al., 2014; Pan et al., 2014). Previous studies have shown that insects with nutrient deficiency present low growth rates, which lead to a small body size and weight loss when entering the adult stage (Hahn, 2005; Zanotto et al., 2010). This was confirmed in the present study (Figure 1), which showed that BPH under nutritional restriction pressures were small in size. However, it is worth noting that their development period was shortened rather than delayed (Figure 2), possibly because different insects respond differently to nutritional deficiency. Similarly, Pan et al. (2014) fed first instar nymphs of BPH with standard artificial feed and amino acid-deficient artificial feed, and found that the development period was significantly shortened, indicating that growth is accelerated when their diets are poor in amino acids. The larval and pupal stages of *Harmonia axyridis* were significantly prolonged after feeding with multiple artificial feed formulations (Li et al., 2020). Proteins and carbohydrates are the two most important basic nutrients for insect growth and development, and provide the necessary amino acids and energy for insect growth and development. Most insects require the best nutritional protein and carbohydrate ratios to reduce the loss of adaptability (Liu et al., 2017). Nutritional deficiency clearly affects insect survival. Long-term nutritional deficiency, in particular, can change biological function and even cause death (Kirk, 2004), and our results also confirm this (Figure 2). Large animals are generally more resistant to nutritional deficiencies than small animals because of their higher metabolic rates and energy reserves (Bauchinger and McWilliams, 2012). For example, body size is positively correlated with resistance to starvation in *Alphitobius diaperinus* and *Anopheles gambiae* (Lehmann et al., 2006; Renault et al., 2010).

Molting and metamorphosis are important for insects (Ishimaru et al., 2016). The energy required for metamorphosis is extracted from the energy stored during larval rearing (Merkey et al., 2011; Wang et al., 2020). Surprisingly, in *Samia cynthia* (Hetz, 2007), *Zophobas rugipes* (Kaiser et al., 2010), and *Apis mellifera* (Schmolz et al., 2005), low energy consumption during metamorphosis was observed. Reduced expression level of genes related to energy metabolism in the BPH or *Sogatella furcifera* was shown to cause phenotypic deformity due to molting difficulty (Yang et al., 2017; Tang et al., 2019). In the present study, the BPH deformity rate also increased with nutrient deficiency, and the differences among the 100, 50, and 25% feed concentrations were significant (Figure 2). We speculate that the energy stored during the larval stage under the 50 and 25% feed treatments was not sufficient to support the molting and metamorphosis of nymphs. Similar results were found for Chrysomelidae and Ephemeroptera species (Aguila et al., 2013).

Nutritional deficiency also affected ovary development in the BPH, as shown by the slow ovarian development, immature eggs, atrophy, and incomplete ovarian morphology (Figure 3). Early nutrition (i.e., larval nutrition) has significant effects on insect phenotype, stress resistance, immune characteristics, energy metabolism, reproduction, and life span (Pan et al., 2014). Previous studies have shown that the energy required for the production of the first nest eggs by adult mosquitoes is entirely derived from the energy accumulated in the larval stage (Telang and Wells, 2004). It has also been shown that a higher ratio of proteins to carbohydrates in food could increase the oviposition of female *Drosophila melanogaster* (Mair et al., 2005; Lee et al., 2008; Skorupa et al., 2008; Jensen et al., 2015).

At the molecular level, the relative expression of *Vg* on the second day of the fourth-instar nymphs was highest in the 25% treatment group. We speculate that this might be related to the length of the nutrition deficiency period, as nutrition deficiency started with third-instar nymphs being fed artificial feed. With the increase in feeding time, nutrition deficiency occurred for longer periods and the expression level of *Vg* was significantly decreased in the low concentration artificial feed group (Figure 4). The Vg protein is synthesized in the fat bodies, secreted into the hemolymph, and then accumulated by cytotoxic action, while yolk protein is formed in the oocyte (Hagedorn and Kunkel, 1979; Raikhel and Dhadialla, 1992; Shen et al., 2019). The tissue expression results showed that the lack of nutrition could inhibit the expression level of the *Vg* gene, further inhibiting the expression of the Vg protein in the ovary, eventually leading to poor ovarian development and less mature eggs (Figures 3, 4).

In addition, our results showed that the total lipid content in BPH fat bodies decreased significantly with a decrease in nutrition (Figure 5), indicating that nutrient restriction directly led to a decrease in total lipid content. In fact, insect fat bodies, comparable to the liver of vertebrates, can store nutrients, detoxify, and provide various biosynthetic metabolites for insect life cycle activities. The size and quantity of fat bodies are affected by the growth and nutrition of insects, and the synthesis and transportation of lipids play an indispensable role in insect fertility (Lu et al., 2016). Fat bodies are the main energy source used in embryo development and 90% of the energy consumed during the embryo development of *Culex quinquefasciatus* is from lipids (Handel, 1993). These lipids are transported to the ovaries of insects, mainly by endocytosis of the Vg protein, through apolipoprotein transport, and some *via* extracellular mechanisms where they need to be hydrolyzed by triglycerides (Ziegler and Antwerpen, 2006; Fruttero et al., 2011; Parra-Peralbo and Culi, 2011). In general, nutritional deficiency can affect the growth and development of BPH, particularly resulting in body size reduction, weight loss, shortened development history, survival decline, and increases in deformity rate. It can also inhibit ovarian development, egg synthesis, *Vg* expression, and lipid synthesis. Actually, BPH feeding can broadly interfere with host physical metabolism and rice plants could also change their metabolic patterns to combat BPH attack and maintain normal biological activities (Alamgir et al., 2016; Kang et al., 2019). Our results were useful to clarify the insect-plant interaction and to provide new resources to control this insect pest.

## Data Availability Statement

The original contributions presented in the study are included in the article/Supplementary Material, further inquiries can be directed to the corresponding author.

## Author Contributions

KK and YC contributed equally to this work. This study was conceived by WZ, KK, YC, and LY performed the experiments. KK analyzed the data and wrote the manuscript. All authors read and approved the final manuscript.

## Funding

This work was financially supported through a grant from the Science and Technology Program of Guizhou Province (Qiankehe foundation-ZK2021-135).

## Conflict of Interest

The authors declare that the research was conducted in the absence of any commercial or financial relationships that could be construed as a potential conflict of interest.

## Publisher’s Note

All claims expressed in this article are solely those of the authors and do not necessarily represent those of their affiliated organizations, or those of the publisher, the editors and the reviewers. Any product that may be evaluated in this article, or claim that may be made by its manufacturer, is not guaranteed or endorsed by the publisher.
